# Weak Singularities of the Isothermal Entropy Change as the Smoking Gun Evidence of Phase Transitions of Mixed-Spin Ising Model on a Decorated Square Lattice in Transverse Field

**DOI:** 10.3390/e23111533

**Published:** 2021-11-18

**Authors:** Jozef Strečka, Katarína Karl’ová

**Affiliations:** Department of Theoretical Physics and Astrophysics, Faculty of Science, P. J. Šafárik University in Košice, Park Angelinum 9, 040 01 Košice, Slovakia; katarina.karlova@upjs.sk

**Keywords:** Ising model, transverse field, exact results, magnetocaloric effect, weak singularity

## Abstract

The magnetocaloric response of the mixed spin-1/2 and spin-*S* (S>1/2) Ising model on a decorated square lattice is thoroughly examined in presence of the transverse magnetic field within the generalized decoration-iteration transformation, which provides an exact mapping relation with an effective spin-1/2 Ising model on a square lattice in a zero magnetic field. Temperature dependencies of the entropy and isothermal entropy change exhibit an outstanding singular behavior in a close neighborhood of temperature-driven continuous phase transitions, which can be additionally tuned by the applied transverse magnetic field. While temperature variations of the entropy display in proximity of the critical temperature $T_{c}$ a striking energy-type singularity $\left(T-T_{c}\right)\log\left|T-T_{c}\right|$, two analogous weak singularities can be encountered in the temperature dependence of the isothermal entropy change. The basic magnetocaloric measurement of the isothermal entropy change may accordingly afford the smoking gun evidence of continuous phase transitions. It is shown that the investigated model predominantly displays the conventional magnetocaloric effect with exception of a small range of moderate temperatures, which contrarily promotes the inverse magnetocaloric effect. It turns out that the temperature range inherent to the inverse magnetocaloric effect is gradually suppressed upon increasing of the spin magnitude *S*.

## 1. Introduction

The magnetocaloric effect (MCE) relates to an adiabatic temperature change of magnetic materials achieved in response to a varying external magnetic field. Although this phenomenon was experimentally detected by Warburg more than a century ago [1], the MCE still remains an active research field in its own right due to its wide application potential in magnetic refrigeration technologies [2,3]. Under the isothermal condition, the raising of the external magnetic field results in the isothermal entropy change $\Delta S_{iso}=S\left(T,H\neq 0\right)-S\left(T,H=0\right)$, whose sign allows one to discern the conventional MCE with $-\Delta S_{iso}>0$ from the inverse MCE with $-\Delta S_{iso}<0$ [4]. The magnetic substances subject to the conventional MCE are heated up (cooled down) under the adiabatic magnetization (demagnetization), while the magnetic entropy is lowered (raised) under the isothermal magnetization (demagnetization). The reverse is of course true for magnetic materials being subject to the inverse MCE.

Most of magnetic materials regrettably show only a rather small magnetocaloric response that considerably prevents their implementation in magnetic refrigeration technologies. From this point of view, spontaneously ordered magnetic materials are expected to be most perspective coolants, because they often exhibit an enhanced MCE in a vicinity of the critical temperature connected to a magnetic order-disorder phase transition between spontaneously ordered and disordered states [5]. It is worthwhile to remark that the spontaneous long-range order may eventually emerge just if the underlying magnetic structure is at least two-dimensional and, hence, the rigorous study of MCE close to the order-disorder phase transitions is beyond the scope of present knowledge due to the incapability to treat the corresponding lattice-statistical spin model by exact means. In spite of its conceptual simplicity, two-dimensional (2D) Ising models are generally not exactly solvable in a nonzero external magnetic field [6].

Interestingly, there exist a few valuable examples of 2D Ising models at least partially accounting for the effect of the external magnetic field, which may bear evidence of the enhanced MCE near the order-disorder phase transition. Among these paradigmatic examples, one could for instance mention Fisher’s superexchange antiferromagnet, which refers to a spin-1/2 Ising model on a decorated square lattice with spatially modulated ferromagnetic and antiferromagnetic interactions in a longitudinal magnetic field [7,8]. Exact solutions for several variants and extensions of the original Fisher’s superexchange model have been found by considering higher spin values, crystal-field anisotropy or different lattice geometries [9,10,11,12,13,14]. A few additional special cases of 2D Ising models partially taking into consideration the longitudinal magnetic field were exactly solved by making use of a precise mapping correspondence with free-fermion 16-vertex or 32-vertex models [15,16,17,18,19,20,21]. One should bear in mind that in all aforementioned 2D Ising models the longitudinal magnetic field was specifically applied only to decorating spins.

Another intriguing open question is whether the enhanced MCE emergent at the order-disorder phase transition is reinforced or contrarily suppressed by quantum fluctuations. This question could be addressed with the help of exactly solved 2D Ising models on a honeycomb lattice [22,23,24] or a decorated square lattice [25,26,27], which take into account a transverse magnetic field introducing into the respective Hamiltonian the transverse component of spin operators that does not commute with the longitudinal one. In our preceding brief report we have convincingly demonstrated that the spin-1/2 Ising model on a decorated square lattice shows in the transverse magnetic field a remarkable crossover between the conventional and inverse MCE [27]. The main focus of the present work is to extend this study through a detailed examination of the entropy and the isothermal entropy change of a mixed spin-1/2 and spin-*S* (S>1/2) Ising model on a decorated square lattice in the transverse magnetic field.

Before doing so, let us make a few comments concerned with an experimental motivation of the proposed theoretical model. At first sight, the considered mixed spin-(1/2, *S*) Ising model on a decorated square lattice might seem to be an artificial toy model, because the applied transverse magnetic field acts exclusively on decorating spins while leaving nodal spins completely unaffected. Although this requirement is not so commonly met in real magnetic materials, however, this peculiar feature may be encountered in magnetic compounds involving magnetic ions with a highly anisotropic *g*-factor (e.g., Dy${}^{3+}$ or Co${}^{2+}$) in combination with magnetic ions with a nearly isotropic *g*-factor (e.g., Cu${}^{2+}$ or Ni${}^{2+}$). The former magnetic ions do not experience the effect of external magnetic field once it is applied perpendicular to a quantization (Ising) axis in contrast with the latter magnetic ions, which are basically influenced by the external magnetic field regardless of its spatial orientation. In this regard, 3d-4f heterobimetallic molecular-based magnetic materials consisting, for instance, from Dy${}^{3+}$ and Cu${}^{2+}$ magnetic ions afford the experimental realization of lattice-statistical spin models with strongly modulated magnetic fields [28,29,30,31,32,33].

**Figure 1:**
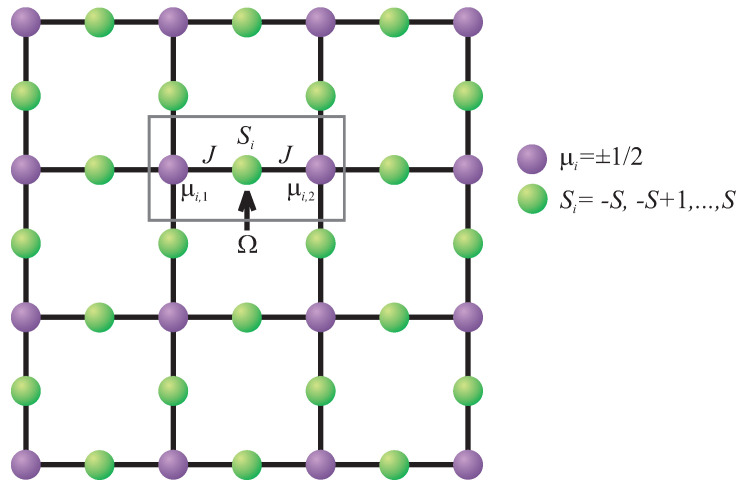
A cross-section from the decorated square lattice. The purple (dark) circles denote lattice positions of the nodal spin-1/2 magnetic ions and the green (light) circles schematically represent lattice positions of the decorating spin-*S* (S>1/2) magnetic ions. A rectangle delimits a three-spin cluster described by the bond Hamiltonian (2).

Furthermore, it is noteworthy that the magnetic structure of the decorated square lattice can be found in four isostructural molecular magnets [M${}^{2+}$(pyrazole)${}_{4}$]${}_{2}$[Nb${}^{4+}$(CN)${}_{8}$] · 4 H${}_{2}$O [34] to be further abbreviated as [M${}_{2}$Nb], which contain some of the following divalent transition-metal ions M${}^{2+}$ = Ni${}^{2+}$, Co${}^{2+}$, Fe${}^{2+}$ or Mn${}^{2+}$. The former two members of this series with either Ni${}^{2+}$ or Co${}^{2+}$ magnetic ions exhibit a spontaneous ferromagnetic ordering in contrast to the latter two members either with Fe${}^{2+}$ or Mn${}^{2+}$ magnetic ions that display a spontaneous ferrimagnetic ordering [34]. The molecular-based magnetic materials [M${}_{2}$Nb] afford an excellent playground for an investigation of the role of a spin magnitude on magnetic properties of the mixed spin-(1/2, *S*) decorated square lattice, which is composed from the spin-1/2 Nb${}^{4+}$ magnetic ions residing nodal sites of a square lattice and the spin-*S* M${}^{2+}$ magnetic ions occupying decorating sites of a square lattice (see Figure 1). A diversity of the available divalent M${}^{2+}$ magnetic ions allows one to tune spin magnitude of the decorating magnetic ions among the spin-1 Ni${}^{2+}$ magnetic ions, the spin-3/2 Co${}^{2+}$ magnetic ions, the spin-2 Fe${}^{2+}$ magnetic ions, or the spin-5/2 Mn${}^{2+}$ magnetic ions [34]. It turns out that the choice of the spin size has significant effect upon the MCE, which is the most intense for the manganese-based analog from this series with the highest spin value [Mn${}^{2+}$(pyrazole)${}_{4}$]${}_{2}$ [Nb${}^{4+}$(CN)${}_{8}$] · 4 H${}_{2}$O [35]. Unfortunately, the molecular-based magnetic materials [M${}_{2}$Nb] cannot be regarded as true experimental representatives of a mixed spin-1/2 and spin-*S* (S>1/2) Ising model on the decorated square lattice, because the spin-1/2 Nb${}^{4+}$ magnetic ions from nodal lattice sites of a square lattice are affected by longitudinal as well as transverse magnetic fields due to their almost isotropic gyromagnetic g-factor [34,35]. In spite of this fact we hope that the results presented in this article could stimulate a targeted design of structural analogs of the molecular-based magnets [M${}_{2}$Nb], which would contain the Ising-like magnetic ions with the highly anisotropic g-factor instead of the nearly isotropic spin-1/2 Nb${}^{4+}$ magnetic ions.

In the present article we will examine basic magnetocaloric properties of a mixed spin-1/2 and spin-*S* (S>1/2) Ising model on the decorated square lattice in the presence of the transverse magnetic field; more specifically, temperature variations of the magnetic entropy, the isothermal entropy change driven by change of the transverse magnetic field and the adiabatic temperature change induced by the transverse magnetic field. It should be pointed out that the investigated mixed spin-1/2 and spin-*S* (S>1/2) Ising model on a decorated square lattice represents a prototypical example of an exactly solved lattice-statistical spin model, which allows a detailed examination of MCE in a vicinity of temperature-driven continuous phase transition additionally tunable by the transverse magnetic field.

## 2. Model and Methods

In this part we will comprehensively examine magnetocaloric properties of the mixed spin-1/2 and spin-*S* (S>1/2) Ising model on a decorated square lattice in the presence of a transverse magnetic field, which is schematically depicted in Figure 1 and defined through the following Hamiltonian:

(1) $${\hat{H}}_{d}=-J\sum_{\langle i,j\rangle }{\hat{S}}_{i}^{z}{\hat{\mu }}_{j}^{z}-\Omega \sum_{i=1}^{2N}{\hat{S}}_{i}^{x}.$$

The Hamiltonian (1) is expressed in terms of the spin-1/2 operators ${\hat{\mu }}_{j}^{z}$ ascribed to the magnetic ions from the nodal lattice sites shown in Figure 1 by purple (dark) circles and the spin-*S* operators ${\hat{S}}_{i}^{z}$ and ${\hat{S}}_{i}^{x}$ ascribed to the magnetic ions from the decorating lattice sites shown in Figure 1 by green (light) circles. The summation symbol 〈i,j〉 runs over the pairs of nearest-neighbor spins and the first term entering into the Hamiltonian (1) thus taking into account the nearest-neighbor interaction *J* between the spin-1/2 and spin-*S* magnetic ions placed at the nodal and decorating lattice sites, respectively, while the second term Ω accounts for the Zeeman’s magnetostatic energy of the decorating spin-*S* magnetic ions in a transverse magnetic field. Finally, *N* denotes the total number of the nodal lattice sites occupied by the spin-1/2 magnetic ions. It is worthwhile to note that the transverse magnetic field Ω introduces into the otherwise classical mixed-spin Ising model given by Equation (1) local quantum fluctuations, which are closely related to the noncommuting character of the spin operators ${\hat{S}}_{i}^{z}$ and ${\hat{S}}_{i}^{x}$ emergent in the interaction and field terms, respectively.

It should be mentioned that the mixed spin-1/2 and spin-*S* (S>1/2) Ising model on a decorated square lattice in a transverse magnetic field has been exactly solved in our previous works [25,26] and, therefore, we will recall just a few basic steps of this exact calculation needed for a determination of magnetocaloric properties not dealt with previously. First, it is useful to rewrite the total Hamiltonian (1) as a sum over the bond Hamiltonians, i.e., ${\hat{H}}_{d}=\sum_{i=1}^{2N}{\hat{H}}_{i}$, where each bond Hamiltonian ${\hat{H}}_{i}$ involves all the interaction terms related to the *i*th decorating spin $S_{i}$ (see Figure 1):

(2) $${\hat{H}}_{i}=-J{\hat{S}}_{i}^{z}\left({\hat{\mu }}_{i,1}^{z}+{\hat{\mu }}_{i,2}^{z}\right)-\Omega {\hat{S}}_{i}^{x}.$$

Owing to the commuting character of the bond Hamiltonians $[{\hat{H}}_{i},{\hat{H}}_{j}]=0$ the partition function of the mixed spin-1/2 and spin-*S* Ising model on a decorated square lattice in a transverse magnetic field can be partially factorized into the following useful form:

(3) $$\begin{array}{c} Z_{d}=\sum_{\left\{\sigma_{i}\right\}}\prod_{i=1}^{2N}{Tr}_{i}\exp\left(-\beta {\hat{H}}_{i}\right), \end{array}$$

where $\beta =1/(k_{B}T)$, $k_{B}$ is Boltzmann’s constant, *T* is the absolute temperature, the symbol $\sum_{\left\{\sigma_{i}\right\}}$ denotes a summation over all available spin configurations of the nodal spin-1/2 magnetic ions and the symbol ${Tr}_{i}$ stands for a trace over degrees of freedom of the *i*th decorating spin-*S* magnetic ion. By employing a trace invariance the latter trace can be obtained after a straightforward diagonalization of the bond Hamiltonian (2) achieved through the canonical spin-rotation transformation:

(4) $$\begin{array}{c} {\hat{S}}_{i}^{x}={\hat{S}}_{i}^{x}{}^{\prime }\cos\phi_{i}+{\hat{S}}_{i}^{z}{}^{\prime }\sin\phi_{i},\;{\hat{S}}_{i}^{z}=-{\hat{S}}_{i}^{x}{}^{\prime }\sin\phi_{i}+{\hat{S}}_{i}^{z}{}^{\prime }\cos\phi_{i}, \end{array}$$

which brings the bond Hamiltonian (2) to the diagonal form when using a suitable choice of the rotation angle $\phi_{i}=\arctan\left[\Omega /J\left(\mu_{i,1}^{z}+\mu_{i,2}^{z}\right)\right]$:

(5) $${\hat{H}}_{i}=-{\hat{S}}_{i}^{z}{}^{\prime }\sqrt{J^{2}{\left(\mu_{i,1}^{z}+\mu_{i,2}^{z}\right)}^{2}+\Omega^{2}}.$$

An explicit form of the Boltzmann weight, which is obtained after tracing out spin degrees of freedom of the *i*th decorating spin-*S* magnetic ion, then reads as follows:

(6) $$\begin{array}{ccc} {Tr}_{i}\exp\left(-\beta {\hat{H}}_{i}\right)\;\; & = & \;\;\sum_{S_{i}^{z}{}^{\prime }=-S}^{S}\exp\left[\beta {\hat{S}}_{i}^{z}{}^{\prime }\sqrt{J^{2}{\left(\mu_{i,1}^{z}+\mu_{i,2}^{z}\right)}^{2}+\Omega^{2}}\right]\\ \;\;\; & = & \;\;\;\sum_{n=-S}^{S}\cosh\left[\beta n\sqrt{J^{2}{\left(\mu_{i,1}^{z}+\mu_{i,2}^{z}\right)}^{2}+\Omega^{2}}\right], \end{array}$$

where *n* is the summation index running over 2S+1 available states of the decorating spin. Obviously, the effective Boltzmann’s weight (6) depends just on two nodal Ising spins ($\mu_{i,1}^{z}$, $\mu_{i,2}^{z}$) and consequently, this expression can be substituted through the generalized decoration-iteration transformation [36,37,38]:

(7) $$\begin{array}{c} {Tr}_{i}\exp\left(-\beta {\hat{H}}_{i}\right)=\sum_{n=-S}^{S}\cosh\left[\beta n\sqrt{J^{2}{\left(\mu_{i,1}^{z}+\mu_{i,2}^{z}\right)}^{2}+\Omega^{2}}\right]=A\exp\left(\beta R\mu_{i,1}^{z}\mu_{i,2}^{z}\right). \end{array}$$

The physical meaning of the decoration-iteration transformation (7) lies in removing all the interaction parameters associated with the *i*th decorating spin-*S* magnetic ion and replacing them by a new unique effective interaction *R* between its two nearest-neighbor nodal spin-1/2 magnetic ions $\mu_{i,1}^{z}$ and $\mu_{i,2}^{z}$. Both unknown mapping parameters *A* and *R* are ’self-consistently’ given by the transformation formula (7), which should remain valid for all four possible spin combinations of two nodal Ising spins $\mu_{i,1}^{z}$ and $\mu_{i,2}^{z}$ providing just two independent equations from the mapping transformation (7). Owing to this fact, two yet unknown mapping parameters *A* and *R* can be unambiguously determined by the following formulas:

(8) $$\begin{array}{c} A={\left(V_{1}V_{2}\right)}^{\frac{1}{2}},\;\beta R=2\ln\left(\frac{V_{1}}{V_{2}}\right), \end{array}$$

which are expressed in terms of two newly defined functions:

(9) $$\begin{array}{c} V_{1}=\sum_{n=-S}^{S}\cosh\left(\beta n\sqrt{J^{2}+\Omega^{2}}\right),\;V_{2}=\sum_{n=-S}^{S}\cosh\left(\beta n\Omega \right). \end{array}$$

At this stage, the decoration-iteration transformation (7) with the mapping parameters *A* and *R* determined by Equations (8) and (9) can be substituted into the right-hand-side of Equation (3) in order to obtain a mapping relation between the partition function $Z_{d}$ of the mixed spin-1/2 and spin-*S* Ising model on a decorated square lattice in a transverse magnetic field and, respectively, the partition function $Z_{0}$ of the spin-1/2 Ising model on a simple square lattice in a zero magnetic field:

(10) $$Z_{d}\left(\beta ,J,\Omega \right)=A^{2N}Z_{0}\left(\beta ,R\right).$$

It is worth noticing that the partition function $Z_{0}$ of the spin-1/2 Ising model on a square lattice in a zero magnetic field is know from the famous Onsager’s exact solution [39]

(11) $$\frac{1}{N}\ln Z_{0}=\ln2+\frac{1}{2\pi^{2}}\int_{0}^{\pi }\int_{0}^{\pi }\ln\left\{\cosh^{2}\left(\frac{\beta R}{2}\right)-\sinh\left(\frac{\beta R}{2}\right)\left[\cos\theta +\cos\phi \right]\right\}d\theta d\phi .$$

From this perspective, the rigorous mapping relationship (10) affords the relevant exact result for the partition function $Z_{d}$ of the mixed spin-1/2 and spin-*S* Ising model on a decorated square lattice in a transverse magnetic field. One of the most important consequences of the Onsager’s exact solution for a spin-1/2 Ising model on a square lattice [39] was rigorous confirmation of a phase transition, which is accompanied with a singular behavior of magnetic and thermodynamic quantities in a vicinity of the critical temperature $\beta_{c}R=R/\left(k_{B}T_{c}\right)=2\ln\left(1+\sqrt{2}\right)$. According to Equation (10), the essential singularity in the partition function $Z_{0}$ of the spin-1/2 Ising model on a square lattice causes the analogous singularity in the partition function Z of the mixed spin-1/2 and spin-*S* Ising model on a decorated square lattice in a transverse magnetic field. It should be pointed out, however, that the nearest-neighbor interaction *R* of the effective spin-1/2 Ising model on a square lattice is not constant, but it depends according to Equations (8) and (9) on the temperature *T*, the coupling constant *J*, the transverse field Ω, as well as, the spin size *S*. Consequently, the critical temperature of the mixed spin-1/2 and spin-*S* Ising model on a decorated square lattice will depend on a relative size of the transverse magnetic field Ω/J and the spin magnitude *S*. The critical temperature of the mixed spin-1/2 and spin-*S* Ising model on a decorated square lattice can be thus simply obtained from a direct comparison of the effective coupling βR given by Equations (8) and (9) with its critical value $\beta_{c}R=2\ln\left(1+\sqrt{2}\right)$, which affords the following critical condition:

(12) $$\sum_{n=-S}^{S}\cosh\left(\beta_{c}n\sqrt{J^{2}+\Omega^{2}}\right)=\left(1+\sqrt{2}\right)\sum_{n=-S}^{S}\cosh\left(\beta_{c}n\Omega \right).$$

The numerical solution of the critical condition (12), e.g., by a bisection method, brings insight into how a relative size of the transverse magnetic field Ω/J and the spin size *S* influence a relative magnitude of the critical temperature $k_{B}T_{c}/J$.

In what follows, our attention will be focused on a detailed examination of basic magnetocaloric characteristics of the mixed spin-1/2 and spin-*S* Ising model on a decorated square lattice, which may exhibit especially pronounced features whenever temperature and the transverse magnetic field drive the investigated spin system close enough to a respective critical point. For this purpose, it is of particular importance to derive from the mapping relation (10) exact expressions for basic thermodynamic quantities such as the Gibbs free energy:

(13) $$G_{d}=-k_{B}T\ln Z_{0}-2Nk_{B}T\ln A$$

and the entropy:

(14) $$\begin{array}{c} S_{d}=k_{B}\ln Z_{0}+\frac{2U_{0}}{RT}\left(\;\sqrt{J^{2}+\Omega^{2}}K_{0}-\Omega K_{1}\;\right)+2Nk_{B}\ln A-\frac{N}{T}\left(\;\sqrt{J^{2}+\Omega^{2}}K_{0}+\Omega K_{1}\;\right). \end{array}$$

For the sake of completeness, let us quote the final expression also for the internal energy $U_{0}$ of the effective spin-1/2 Ising model on a square lattice derived by Onsager [39] in the thermodynamic limit N→∞:

(15) $$\begin{array}{c} \frac{U_{0}}{NR}=-\frac{1}{4}\coth\left(\frac{\beta R}{2}\right)\left\{1+\frac{2}{\pi }\left[2\tanh^{2}\left(\frac{\beta R}{2}\right)-1\right]K\left(z\right)\right\}, \end{array}$$

where K(z) is the complete elliptic integral of the first kind with the modulus *z*:

(16) $$\begin{array}{c} K\left(z\right)=\int_{0}^{\pi /2}\frac{1}{\sqrt{1-z^{2}\sin^{2}\theta }}d\theta ,\;z=\frac{2\sinh\left(\frac{\beta R}{2}\right)}{\cosh^{2}\left(\frac{\beta R}{2}\right)}, \end{array}$$

and temperature-dependent coefficients $K_{0}$ and $K_{1}$ emergent in Equation (14) are given by:

(17) $$\begin{array}{c} K_{0}=\frac{\sum_{n=-S}^{S}n\sinh\left(\beta n\sqrt{J^{2}+\Omega^{2}}\right)}{\sum_{n=-S}^{S}\cosh\left(\beta n\sqrt{J^{2}+\Omega^{2}}\right)},\;K_{1}=\frac{\sum_{n=-S}^{S}n\sinh\left(\beta n\Omega \right)}{\sum_{n=-S}^{S}\cosh\left(\beta n\Omega \right)}. \end{array}$$

The derived exact analytical formulas (14)–(17) allow a straightforward computation of the basic magnetocaloric properties as, for instance, the isothermal entropy change or the adiabatic temperature change. The isothermal entropy change can be calculated as a difference of the entropy at nonzero and zero magnetic fields $\Delta S_{iso}=S_{d}\left(T,\Omega \neq 0\right)-S_{d}\left(T,\Omega =0\right)$ at the constant temperature, while the adiabatic change of temperature can be traced back from contour lines of the density plot of the entropy (14) in the field-temperature plane.

## 3. Results and Discussion

Before proceeding to a discussion of the most interesting results for the magnetocaloric properties of the mixed spin-1/2 and spin-*S* Ising model on a decorated square lattice in a transverse magnetic field defined through the Hamiltonian (1), it is worthwhile to remark that the finite-temperature phase diagrams, the longitudinal and transverse magnetizations, the specific heat and susceptibility were extensively examined in our two preceding articles [25,26], to which readers interested in a more comprehensive understanding of temperature behavior of these magnetic and thermodynamic quantities are referred to. On the other hand, basic magnetocaloric characteristics of the mixed spin-1/2 and spin-*S* Ising model on a decorated square lattice in a transverse magnetic field have not been dealt with previously except the particular case with the spin value S=1/2 [27]. Our recent brief report concerned with this particular case has convincingly evidenced that the spin-1/2 Ising model on a decorated square lattice displays an anomalous magnetocaloric response in vicinity of a continuous phase transition, which can be driven either by temperature or the transverse magnetic field [27]. In the present article we will therefore resort to a systematic investigation of the magnetocaloric properties of the mixed spin-1/2 and spin-*S* Ising model on a decorated square lattice in a transverse magnetic field when comparing basic magnetocaloric features such as entropy, isothermal entropy change and adiabatic temperature change for four selected spin values S=1, 3/2, 2 and 5/2 of the decorating atoms.

**Figure 2:**
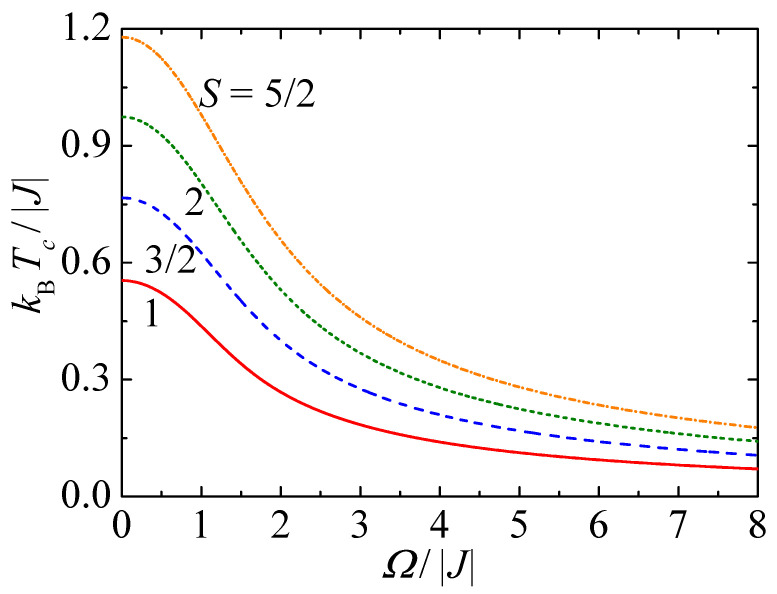
Finite-temperature phase diagrams of the mixed spin-1/2 and spin-*S* Ising model on a decorated square lattice in the form of plots the critical temperature $k_{B}T_{c}/\left|J\right|$ versus the transverse magnetic field Ω/|J| for four selected values of the spin magnitude S=1, 3/2, 2 and 5/2.

Let us begin with the finite-temperature phase diagrams of the mixed spin-1/2 and spin-*S* (S>1/2) Ising model on a decorated square lattice, which are depicted in Figure 2 for four selected values of the spin magnitude *S* in the form of plots the critical temperature $k_{B}T_{c}/\left|J\right|$ versus the transverse magnetic field Ω/|J|. It is worth mentioning that the critical temperature was calculated according to the critical condition (12), which is invariant with respect to a sign change of the coupling constant J→−J implying the identical critical behavior of the ferromagnetic (J>0) and ferrimagnetic (J<0) model system. The lines displayed in Figure 2 bring insight into the functional dependence of the dimensionless critical temperature $k_{B}T_{c}/\left|J\right|$ on a relative size of the transverse magnetic field Ω/|J|. Note that the ferromagnetic (ferrimagnetic) ordering with a non-zero spontaneous magnetization is realized below the depicted phase boundaries for J>0(J<0), while above them the disordered paramagnetic phase with zero spontaneous magnetization takes place. As one can see from Figure 2, the critical temperature acquires its maximal value in the zero-field limit from which it monotonously decreases upon strengthening of the transverse magnetic field until it tends to zero in the asymptotic limit Ω/|J|→∞. This statement holds true for any spin value *S*, whereby the rise of spin magnitude merely shifts the critical temperature to higher values. The monotonous decline of the critical temperature observable upon increasing of the transverse magnetic field can be attributed to a strengthening of local quantum fluctuations, because the transverse field exclusively acting on the decorating spins promotes a quantum superposition of all their available spin states.

**Figure 3:**
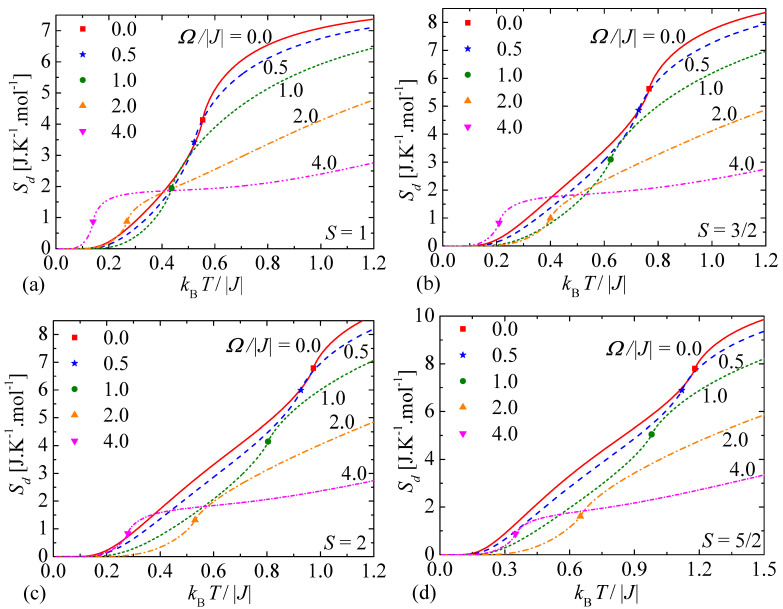
Temperature variations of the molar entropy of the mixed spin-1/2 and spin-*S* (S>1/2) Ising model on a decorated square lattice for a few different values of the transverse magnetic field and four selected values of the spin magnitude: (**a**) S=1; (**b**) S=3/2; (**c**) S=2; (**d**) S=5/2. Filled symbols of different styles allocate singular points of the type $\propto \left(T-T_{c}\right)\log\left|T-T_{c}\right|$.

**Figure 4:**
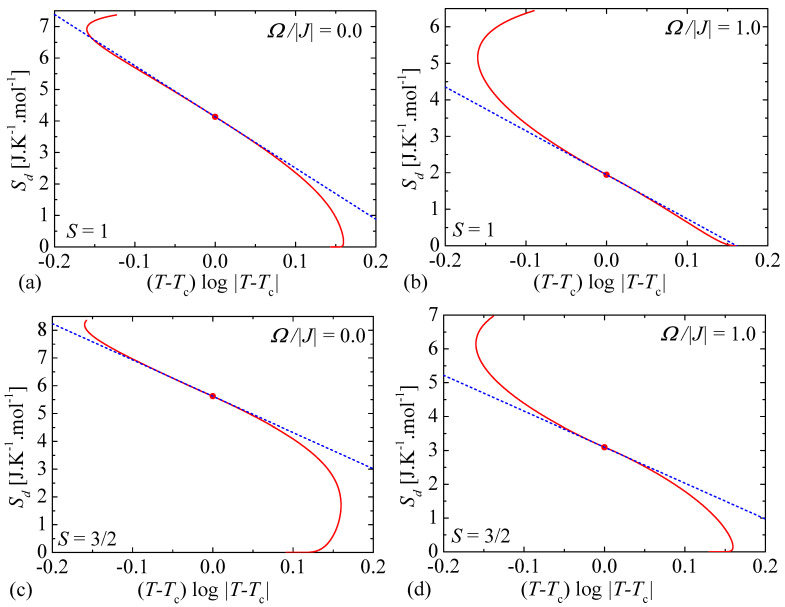
The molar entropy of the mixed spin-1/2 and spin-*S* (S>1/2) Ising model on a decorated square lattice as a function of the temperature deviation $\left(T-T_{c}\right)\log\left|T-T_{c}\right|$ from its critical value for two different values of the spin magnitude and transverse magnetic fields: (**a**) S=1, Ω/|J|=0.0; (**b**) S=1, Ω/|J|=1.0; (**c**) S=3/2, Ω/|J|=0.0; (**d**) S=3/2, Ω/|J|=1.0. Blue broken lines are linear fits of the respective dependencies, which prove a singular character of the entropy that is in a close vicinity of the critical points (filled red circles) proportional to $\propto \left(T-T_{c}\right)\log\left|T-T_{c}\right|$.

Typical temperature dependencies of the molar entropy of the mixed spin-1/2 and spin-*S* (S>1/2) Ising model on a decorated square lattice are shown in Figure 3 for a few different values of the transverse magnetic field and four selected values of the spin magnitude *S*. The entropy monotonously increases with the increasing of temperature, whereby the largest increment of the entropy can be observed in the proximity of a continuous order-disorder phase transition that is accompanied with a weak singularity of the type $\propto \left(T-T_{c}\right)\log\left|T-T_{c}\right|$ visualized in Figure 3 by different filled symbols. To shed light on a singular character of the respective critical points we have plotted in Figure 4 the entropy against the temperature deviation $\left(T-T_{c}\right)\log\left|T-T_{c}\right|$ from its critical value for two selected values of the spin magnitude and two different values of the transverse magnetic field. It is obvious from Figure 4 that the entropy generally displays in a close vicinity of the critical temperature a linear dependence when it is plotted against the temperature deviation $\left(T-T_{c}\right)\log\left|T-T_{c}\right|$. This fact convincingly evidences that the entropy exhibits close to the critical temperature the weak singularity of the type $\left(T-T_{c}\right)\log\left|T-T_{c}\right|$, which arises according to Equation (14) from the respective singularity of the internal energy $U_{0}$ of the effective spin-1/2 Ising model on a square lattice (15).

Let us return back to a detailed analysis of temperature dependencies of the entropy (Figure 3) from the perspective of the MCE. The temperatures, at which the entropy dependencies calculated at non-zero transverse magnetic field (broken lines) cross zero-field dependence of the entropy (solid line), represent crossover points determining a change of the conventional MCE to the inverse MCE or vice versa. The mixed spin-1/2 and spin-*S* (S>1/2) Ising model on a decorated square lattice predominantly exhibits the conventional MCE, while the inverse MCE can be mostly detected at low enough temperatures and sufficiently high transverse magnetic fields (e.g., Ω/|J|=4.0) where the entropy at non-zero transverse fields overwhelms over the zero-field entropy. It also follows from comparison of Figure 3a–d that the parameter region pertinent to the inverse MCE gradually diminishes upon increasing of the spin size. However, two crossover temperatures observable in temperature dependencies of the entropy for relatively small transverse magnetic field Ω/|J|=0.5 allocate another rather narrow temperature range inherent to the inverse MCE, whereby the lower crossover temperature occurs close to a continuous phase transition for the given transverse magnetic field (e.g., Ω/|J|=0.5) and the upper one emerges near a continuous phase transition corresponding to the zero field Ω/|J|=0.0 [see Figure 3a–d]. It could be thus concluded that a relative strength of the transverse magnetic field basically determines the number as well as position of the crossover points between the conventional and inverse MCE, namely, one may for instance detect for sufficiently large transverse magnetic field Ω/|J|=4.0 only one crossover temperature for the spin magnitudes S=1 and S=3/2 [Figure 3a,b], two crossover temperatures for S=2 [Figure 3c], or any crossover temperature for S=5/2 [Figure 3d].

**Figure 5:**
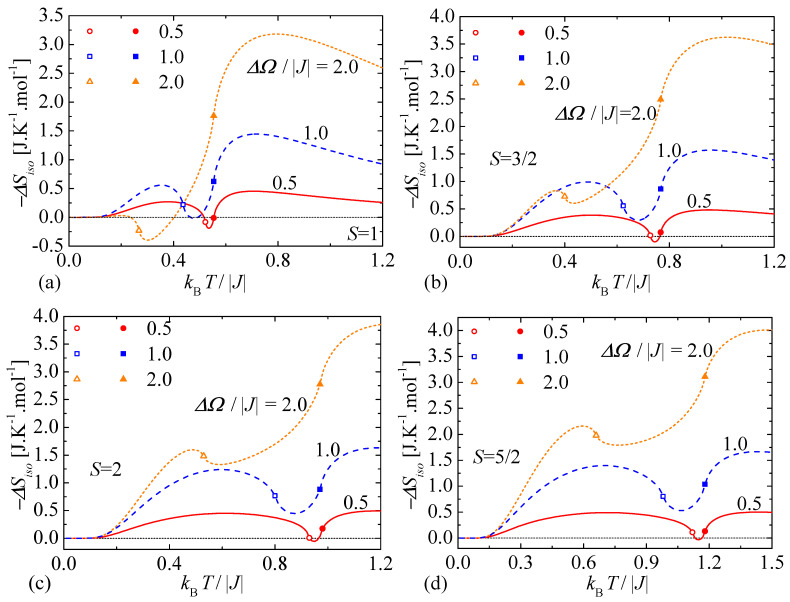
Temperature dependencies of the isothermal entropy change for three different values of the transverse-field change ΔΩ/|J|=0.5,1.0 and 2.0 and four selected spin magnitude: (**a**) S=1; (**b**) S=3/2; (**c**) S=2; (**d**) S=5/2. Thin dotted line at $-\Delta S_{iso}=0$ is only guide for eyes, which enables to distinguish the conventional and inverse MCE. Open symbols denote weak singularities located at critical points of continuous phase transitions at the transverse magnetic fields Ω/|J|=0.5,1.0 and 2.0, while filled symbols mark weak singularities of the zero-field entropy emergent at the critical temperature: (**a**) $k_{B}T/\left|J\right|\approx 0.554$; (**b**) $k_{B}T/\left|J\right|\approx 0.767$; (**c**) $k_{B}T/\left|J\right|\approx 0.970$; (**d**) $k_{B}T/\left|J\right|\approx 1.180$.

Even more direct evidence of the crossover between the conventional and inverse MCE can be gained from a detailed analysis of the isothermal entropy change $-\Delta S_{iso}\left(T,\Delta \Omega \right)=S_{d}\left(T,\Omega =0\right)-S_{d}\left(T,\Omega \neq 0\right)$, which characterizes a basic caloric response of the investigated spin system with respect to a variation of the transverse magnetic field as it determines amount of the heat exchanged with an environment during the isothermal process. Temperature dependencies of the isothermal entropy change of the mixed spin-1/2 and spin-*S* Ising model on a decorated square lattice are depicted in Figure 5 for three different values of the transverse-field change ΔΩ/|J| and four selected values of the spin magnitude *S*. It is worthwhile to recall that a positive value of the isothermal entropy change $-\Delta S_{iso}>0$ implies the conventional MCE, while the negative isothermal entropy change $-\Delta S_{iso}<0$ indicates the inverse MCE. The temperature dependencies of the isothermal entropy change plotted in Figure 5 thus serve in evidence that the mixed spin-1/2 and spin-*S* Ising model on a decorated square lattice predominantly shows the conventional MCE and the inverse MCE can be detected only in a rather limited range of temperatures and magnetic fields. In general, the isothermal entropy change displays a marked temperature dependence when it rises steadily in a low-temperature region from zero value until it reaches a round local maximum, then it drops down to a more or less shallow local minimum detectable at moderate temperatures before it finally shows another round maximum in a high-temperature region. The outstanding and highly non-monotonic temperature dependence of the isothermal entropy change additionally contains two weak singularities of the type $\propto \left(T-T_{c}\right)\log\left|T-T_{c}\right|$, which are highlighted in Figure 5 by different empty and filled symbols surrounding the local minimum. Finally, let us make a few comments on an existence of the inverse MCE. The inverse MCE arises just in a rather narrow range of temperatures $k_{B}T/\left|J\right|\in \left(0.517;0.554\right)$ when considering the spin magnitude S=1 and the relatively low magnetic-field change ΔΩ/|J|=0.5, inside of which the isothermal entropy change achieves its local minimum $-\Delta S_{iso}\approx -0.190$ J.K${}^{-1}$.mol${}^{-1}$ located in between two weak singularities, see Figure 5a. It directly follows from Figure 5b–d that the inverse MCE at the same magnetic-field change ΔΩ/|J|=0.5 shifts to higher temperatures upon increasing of the spin magnitude *S* and it is simultaneously limited to a narrower interval of temperatures. Beside this, the inverse MCE can be detected at higher values of the magnetic-field change as for instance corroborated by the dependence shown in Figure 5a for the spin magnitude S=1 and ΔΩ/|J|=2.0.

**Figure 6:**
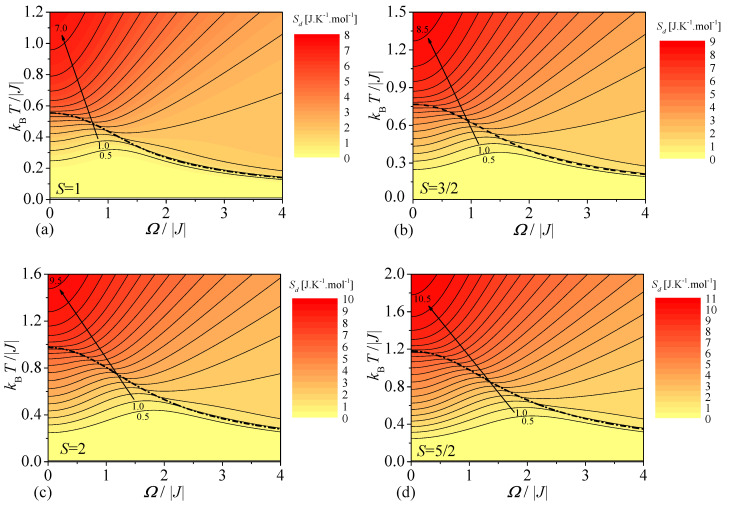
Density plots of the molar entropy of the mixed spin-1/2 and spin-*S* Ising model on a decorated square lattice in the plane transverse magnetic field versus temperature for four selected values of the decorated spins: (**a**) S=1; (**b**) S=3/2; (**c**) S=2; (**d**) S=5/2. Broken lines show dependence of the critical temperature on the transverse magnetic field.

The MCE of the mixed spin-1/2 and spin-*S* Ising model on a decorated square lattice can be also studied through the adiabatic change of temperature induced upon variation of the transverse magnetic field. To this end, the density plots of the molar entropy are displayed in Figure 6 in the transverse magnetic field versus temperature plane, whereby the relevant contour lines determine the adiabatic changes of temperature under the isoentropic magnetization or demagnetization. The highest density of the isoentropy lines can be observed independently of the spin size *S* around the phase boundaries (thick broken lines), which determine continuous order-disorder phase transitions between the spontaneously ordered ferromagnetic (ferrimagnetic) state and the disordered paramagnetic state. It is obvious from Figure 6a that the mixed spin-1/2 and spin-1 Ising model on a decorated square lattice shows temperature rise upon lowering of the transverse field up to Ω/|J|≈1, which is successively followed by temperature lowering whenever the entropy is sufficiently small $S_{d}\leq 1.0\;J\cdot K\cdot {\mathrm{mol}}^{-1}$. The qualitatively analogous behavior can be also found for the mixed spin-1/2 and spin-*S* Ising model on a decorated square lattice with other particular spin values S=3/2,2 and 5/2, which merely shift the relevant temperature decline towards higher values of the transverse magnetic field. If a small enough entropy $S_{d}\leq 1.0\;J\cdot K\cdot {\mathrm{mol}}^{-1}$ is considered, the temperature of the mixed spin-1/2 and spin-5/2 Ising model on a decorated square lattice already starts to decrease at higher transverse magnetic fields Ω/|J|≲2 under the adiabatic demagnetization, see Figure 6d. If the entropy is higher than the value $S_{d}\geq 1.5\;J\cdot K\cdot {\mathrm{mol}}^{-1}$, then the temperature decreases upon lowering of the transverse field in a high-field regime, after which it shows a gentle rise in a vicinity of the critical field, before it finally shows a gradual temperature decline in a low-field regime. For completeness, it worthy to note that the temperature may monotonously decrease to a certain finite value upon lowering of the transverse magnetic field provided that the entropy exceeds the critical value corresponding to the order-disorder phase transition at zero field.

## 4. Conclusions

In the present article we have investigated in detail the basic magnetocaloric properties of the mixed spin-1/2 and spin-*S* (S>1/2) Ising model on a decorated square lattice in a transverse magnetic field, which was exactly solved through a rigorous mapping correspondence with the zero-field Ising square lattice established with the help of generalized decoration-iteration transformation. Temperature dependencies of the entropy, the isothermal entropy change and the adiabatic temperature change were comprehensively explored depending on the transverse magnetic field and the spin magnitude *S*. It has been demonstrated that the mixed spin-1/2 and spin-S Ising model on a decorated square lattice predominantly exhibits the conventional MCE, while the inverse MCE contrarily appears only rarely and it is further suppressed by increasing spin magnitude *S*. The inverse MCE emerges either in a relatively narrow temperature range around the critical temperature or under the specific combination of sufficiently low temperatures and high enough transverse magnetic fields.

It should be mentioned that temperature dependencies of the entropy of the mixed spin-1/2 and spin-*S* Ising model on a decorated square lattice display a peculiar weak singularity $\left(T-T_{c}\right)\log\left|T-T_{c}\right|$, which emerges at the critical temperature of the continuous order-disorder phase transition. Similarly, temperature dependencies of the isothermal entropy change contain two weak singularities of the same type $\left(T-T_{c}\right)\log\left|T-T_{c}\right|$, which delimit the temperature range involving a local minimum of the isothermal entropy change. These two singularities appear at the critical temperatures, which correspond to the initial and final transverse magnetic field determining the respective magnetic-field change. Lastly, it has been demonstrated that the mixed spin-1/2 and spin-*S* Ising model on a decorated square lattice exhibits a monotonic decline of temperature during the adiabatic demagnetization whenever the entropy is sufficiently high, while temperature first rises and then decreases under the adiabatic demagnetization if the entropy is fixed at a low enough value.

## Data Availability

The data presented in this study are available upon reasonable request from the authors.

## Abbreviations

The following abbreviations are used in this manuscript:

MCE	Magnetocaloric effect
2D	Two-dimensional
